# Evaluation of O2PLS in Omics data integration

**DOI:** 10.1186/s12859-015-0854-z

**Published:** 2016-01-20

**Authors:** Said el Bouhaddani, Jeanine Houwing-Duistermaat, Perttu Salo, Markus Perola, Geurt Jongbloed, Hae-Won Uh

**Affiliations:** 1 Department of Medical Statistics and Bioinformatics, LUMC, Albinusdreef 2, Leiden, 2300 RC The Netherlands; 2 National Institute for Health and Welfare (THL), Mannerheimintie 166, Helsinki, FI-00271 Finland; 3 Department of Statistics, EEMCS, TU Delft, Mekelweg 4, Delft, 2628 CD The Netherlands

**Keywords:** Integration of Omics data, Dimension reduction, Latent variable regression, O2PLS

## Abstract

**Background:**

Rapid computational and technological developments made large amounts of omics data available in different biological levels. It is becoming clear that simultaneous data analysis methods are needed for better interpretation and understanding of the underlying systems biology. Different methods have been proposed for this task, among them Partial Least Squares (PLS) related methods. To also deal with orthogonal variation, systematic variation in the data unrelated to one another, we consider the Two-way Orthogonal PLS (O2PLS): an integrative data analysis method which is capable of modeling systematic variation, while providing more parsimonious models aiding interpretation.

**Results:**

A simulation study to assess the performance of O2PLS showed positive results in both low and higher dimensions. More noise (50 % of the data) only affected the systematic part estimates. A data analysis was conducted using data on metabolomics and transcriptomics from a large Finnish cohort (DILGOM). A previous sequential study, using the same data, showed significant correlations between the Lipo-Leukocyte (LL) module and lipoprotein metabolites. The O2PLS results were in agreement with these findings, identifying almost the same set of co-varying variables. Moreover, our integrative approach identified other associative genes and metabolites, while taking into account systematic variation in the data. Including orthogonal components enhanced overall fit, but the orthogonal variation was difficult to interpret.

**Conclusions:**

Simulations showed that the O2PLS estimates were close to the true parameters in both low and higher dimensions. In the presence of more noise (50 %), the orthogonal part estimates could not distinguish well between joint and unique variation. The joint estimates were not systematically affected. Simultaneous analysis with O2PLS on metabolome and transcriptome data showed that the LL module, together with VLDL and HDL metabolites, were important for the metabolomic and transcriptomic relation. This is in agreement with an earlier study. In addition more gene expression and metabolites are identified being important for the joint covariation.

## Background

With rapid and continuous technological improvements large amounts of omics data from different levels (genome, transcriptome, proteome and metabolome) are now available. In an integrative systems biology approach, it is becoming increasingly clear that the integration of omics data will provide a better understanding of biological systems. Towards this end, the simultaneous analysis of two data sets is an important task to better understand the relationships between different biological functional levels.

Statistically, integrative approaches face theoretical and computational issues: the typical “large p, small n” problem as in high dimensional data. Some statistical methods require the inverse of matrices; often they are singular, this can be dealt with by penalization or dimension reduction. Interpretation of the results of the analysis is yet another major challenge. In terms of integrating two data sets the following questions need to be answered: (i) which variables in one data set are related to those in another data set, (ii) which variables are not related, but still important, in each of the data sets, and (iii) which variables are relevant, i.e. provide more insight into the biological systems?

A statistical solution is to perform variable selection while combining the two types of variables in the modeled integration process: for example, a regularized version of canonical correlation analysis (CCA) [1], and a variant of partial least squares (PLS) regression [2] called sparse PLS [3] to simultaneously integrate and select variables using lasso penalization [4].

**Fig. 1:**
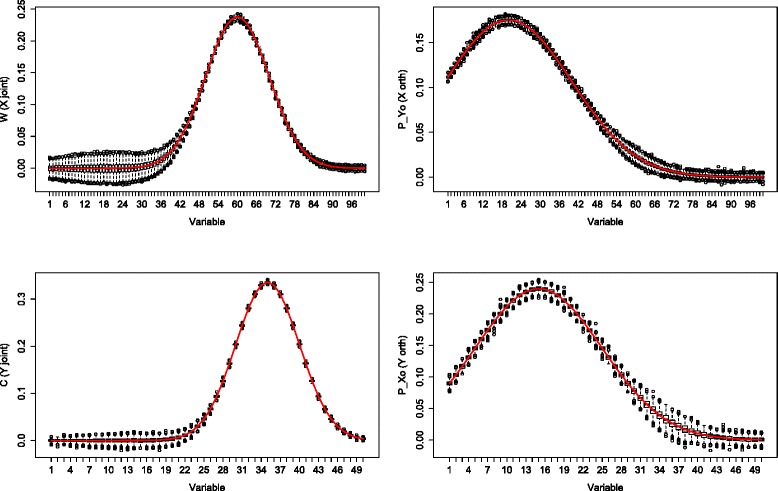
Simulation: low dimensions little noise. Boxplots of 1000 simulations in which *X* (*upper* row) contains 500 samples and 100 variables, *Y* (*lower* row) contains 500 samples and 50 variables. Noise contributed for 5 % of the total variation. The first column corresponds to the joint part, the second column depicts the orthogonal part. The red line denotes the true loading profile

The integration and the variable selection of two different types of omics data sets is nowadays an active research subject. For example, Inouye et al. [5] assessed metabonomic, transcriptomic, and genomic variation for a large population-based cohort from the capital region of Finland. For an overview of the data integration and the different analyses in the study we refer to Figure 1 of their paper [5]. In this work we focus on the first part of data integration of the paper: ‘metabolite associations of gene modules’. First they identified the sets of highly correlated genes, such as the lipid-leukocyte (LL) module, using network analysis of the transcriptomic data. Next a Spearman’s rank correlation was used to identify fine-scale detail of potentially causative/reactive effects between the LL module expression profile (defined by its first principal component) and the individual metabolites. The motivation of the present paper lies in this sequential analysis procedure. In other areas of biostatistics, simultaneous joint modeling of the variables is known to be more efficient than analyzing data sequentially: network construction, identifying the latent variable or module, and correlating this identified module with the individual metabolites.

Model estimates for integrative parts in the data are often not representing the true underlying biological relation when systematic variation unrelated to the outcome is present, the estimates are biased due to this variation. It has been demonstrated that PLS suffers from this [6]. To deal with this, extensions of PLS have been developed. The asymmetric Orthogonal PLS (OPLS) [7], tries to correct for systematic variation in the design matrix before presenting the data to PLS. The main advantage is an easier interpretation of the model: the model estimates focus more on the predictive variation in the design matrix. In order to integrate two data sets, we need a symmetric approach of OPLS. The Two-way Orthogonal PLS (O2PLS) model [6] is a symmetric method, modeling both predictive and systematic variation. The model decomposes the variation present in two data matrices, for example two omics data matrices *X* and *Y*, into three parts. In the first joint part, underlying latent variables in both data matrices are assumed to induce the relationship between *X* and *Y*. This joint part can be seen as a representation of the integration of the two data sets. The second part is called the orthogonal part. Underlying latent variables, independent from those in the joint part, are assumed to be responsible for the unique systematic variation in *X* (*Y*), which does not contribute to the prediction of *Y* (*X*). The third part indicates the noise part, and captures the unsystematic variation in the data.

The aim of this paper is twofold. Our first aim is to jointly model metabolomics and transcriptomics data, in the light of previous study by Inouye et al. [5], to gain a better insight in the interplay between the two omics by decomposing the data in three parts. We extract latent variables for the joint and orthogonal part, and summarize relevant information by looking at the amount of variation captured by these latent variables. Our second aim is to investigate the performance of the O2PLS estimates, in terms of accuracy, with a simulation study under different conditions. We will look at the accuracy in terms of bias, using settings similar to those present in real metabolomics and transcriptomics data.

Integrating metabolomics and transcriptomics using O2PLS is not new. A small scale integration, on 12 aspen grown in a controlled environment, of 453 metabolomic variables and 27,648 transcriptomic data has been performed in [8]. Our analysis is in a larger scale, namely human epidemiological study, consisting of 466 participants. In the metabolomics data set (containing 137 metabolites) we have a classical situation of more participants than variables; the transcriptomics data contains more variables (35419) than participants.

This paper is organized as follows: the “Methods” Section discusses the symmetric integration method O2PLS. A simulation study is set up to assess its performance. In the “Results” Section the simulation results are discussed, furthermore metabolomics and transcriptomics data are analyzed with O2PLS. The “Discussion” Section gives an interpretation of the results from the simulations and data analysis, as well as commenting on the O2PLS model and arguing for a probabilistic approach.

## Methods

### Previous methods

The Partial Least Squares (PLS) method was introduced by Wold [2] to project a *centered* design matrix *X* to a lower dimensional latent variable space:

(1) $$ X = T P^{\mathrm{T}} + E.  $$

Here *T* contains the lower dimensional data. The matrix *P* contains the directions in the *X* space which optimizes the covariance *T*^T^*Y* (where *Y* has zero mean). The matrix *TP*^T^ is to be seen as a ‘best’ approximation of *X* based on the covariance with *Y*. The proof for this is deferred to a separate paragraph later on in this section. The matrix *E* contains the residuals.

The PLS method is a popular method in chemometrics, and from this area an extension was proposed to deal with orthogonal variation: variation important for *X* but unrelated to *X*. This method was named Orthogonal PLS [7]:

(2) $$ X = \tilde{T} \tilde{P}^{\mathrm{T}} + T_{\bot} P_{\bot}^{\mathrm{T}} + \tilde{E}.  $$

Again $\tilde {T}\tilde {P}^{\mathrm {T}}$ represents a best approximation based on the covariance with *Y*, but the direction vectors in $\tilde {P}$ are corrected for (i.e. do not contain directions of) orthogonal variation. The orthogonal variation in *X* is approximated with $T_{\bot } P_{\bot }^{\mathrm {T}}$.

Both PLS and OPLS deal with outcome vectors. While generalizations can be made to make them suitable for an outcome matrix, they focus on regressing *Y* on *X*, but not simultaneously the other way around. This symmetric approach is appropriate for integrating multiple omics data, while also prediction in both ways can be done.

### The O2PLS model

The Two-way Orthogonal PLS (O2PLS) model [6] is a symmetric method capable of dealing with systematic variation. It is a generalization of PLS, correcting for orthogonal variation in both data matrices *X* and *Y*. The model decomposes the variation in the two data matrices into a joint, orthogonal and noise part. The model assumes that some underlying unobservable latent variables are responsible for the variation in the joint and orthogonal part. Define the number of joint latent variables as *a*. The number of *X*-components that are orthogonal to *Y* is denoted by *n*_*x*_. The number of *Y*-components that are orthogonal to *X* is denoted by *n*_*y*_. Let *X* be *N*×*p* and *Y* be *N*×*q*. The O2PLS model can be seen as a factor analysis model:

(3) $$ \begin{aligned} X & = TW^{\mathrm{T}} + T_{\textit{Y}\bot}P_{\textit{Y}\bot}^{\mathrm{T}} + E \\ Y & = UC^{\mathrm{T}} + U_{\textit{X}\bot}P_{\textit{X}\bot}^{\mathrm{T}} + F \\ \end{aligned}  $$

The inner relations for approximating *Y* with *X* and vice versa are

(4) $$ \begin{aligned} U & = TB_{T} + H \\ T & = UB_{U} + \tilde{H} \\ \end{aligned}  $$

In this model the *scores* are

(5) $$ T (N\times a), T_{\textit{Y}\bot} (N\times n_{x}), U (N\times a), U_{\textit{X}\bot} (N\times n_{y}).  $$

They represent a projection of the observed data *X* and *Y* to a lower dimensional ‘optimal’ subspace. The *loadings* are

(6) $$ W (p\times a), C (p\times a), P_{\textit{Y}\bot} (p\times n_{x}), P_{\textit{X}\bot} (p\times n_{y}),  $$

and they assign ‘importance’ to each *X* and *Y* variable to the corresponding subspace. The *noise matrices* are

(7) $$ E (N\times p), F (N\times q), H (N\times a), H' (N \times a).  $$

They capture all ‘left over’ variation not captured by the scores.

To approximate *Y* with *X* (or *X* with *Y*), we need the corresponding inner relation defined via *B*_*T*_ (or *B*_*U*_) in (4). A description of the O2PLS algorithm can be found in Trygg’s paper [6]. The inner relation can be recognized as being an ordinary linear model.

The optimal number of latent variables (*a*, *n*_*X*_, *n*_*Y*_) are in the ideal situation known a priori. In practice this is rare, and a cross-validation (CV) procedure is often used. However, given the large number of variables in the transcriptome and the three dimensional space in which optimization takes place, the CV procedure quickly becomes cumbersome. Hence an alternative method is proposed: we base our cross validation criterion partially on the mean squared error prediction, and moreover on the coefficients of determination (*R*^2^) of the inner relation fit (4), since correcting for orthogonal variation usually improves the fit of the inner relation regression (4) up to a certain number of orthogonal components. The procedure can be summarized as follows:

1. We choose a vector of values for the number of joint components *a*.
2. For *fixeda* we choose the number of orthogonal components *n*_*X*_ and *n*_*Y*_ that maximize the sum of the two coefficients of determination (*R*^2^) of the inner relation regression (4). Mathematically: we search in a two dimensional grid the integers *n*_*X*_ and *n*_*Y*_ that maximize
  (8) $$ (n_{X}, n_{Y}) \mapsto 1-\frac{\sum (H_{UT})_{i,j}^{2}}{\sum U_{i,j}^{2}} + 1-\frac{\sum (H_{TU})_{i,j}^{2}}{\sum T_{i,j}^{2}}.  $$
  We also consider the value zero for the number of orthogonal parts.
3. Two Mean Squared Errors (MSE) of Prediction -concerning $\sum (\hat {Y}-Y)^{2}$ and $\sum (\hat {X}-X)^{2}$ - are calculated with 10-fold cross-validation to determine *a* with the previously obtained *n*_*X*_ and *n*_*Y*_ fixed.
4. We go back to step 2 using for *a* the next element in the vector of values as chosen in step 1.

The quality of the O2PLS estimates depends on the accuracy of the estimated covariance matrix *S*=*X*^T^*Y*. Suppose *X*=*E* and *Y*=*F*, so *X* and *Y* are only noise. The covariance matrix *S* can be decomposed with SVD: *S*=*WDC*^T^, where *W* and *C* are unit norm. It may be that we will observe a ‘large’ positive loading value, since the norm of the loading vectors are forced to be one, and may mistakenly conclude that *X* and *Y* are related. However since *X* and *Y* are independent the projected data *T* and *U* are little correlated (due to noisy variation), thus the inner relation parameters *B*_*T*_ and *B*_*U*_ will have a small magnitude.

Orthogonal correction captures variation unrelated to the joint part. The residual data is hoped to correlate stronger, thus providing a better inner relation fit. Especially with a high number of variables, this may improve the fit (and thus interpretability of the obtained loadings) substantially. Estimation accuracy will not likely be improved by correcting for orthogonal variation, since we do not add information concerning the relation between *X* and *Y*. However the exact statistical implications of orthogonality correction on the joint part estimators is still an unclear matter.

### Proof of optimality

To make clear why the singular value decomposition is important for O2PLS, some optimality properties are proven.

The joint part maximizes the covariance between the joint scores *u*=*Yc* and *t*=*Xw*:

(9) $$ u^{\mathrm{T}} t = c^{\mathrm{T}} Y^{\mathrm{T}} X w.  $$

The maximization is over the set $\{w\in \mathbb {R}^{p},c\in \mathbb {R}^{q} : w^{\mathrm {T}} w = c^{\mathrm {T}} c = 1\}$. Suppose $C_{Y} D W_{X}^{\mathrm {T}}$ is a singular value decomposition of *Y*^T^*X*, where *C*_*Y*_ is *q*×*q*, *D* is *q*×*p* and *W*_*X*_ is *p*×*p*. Then the objective function becomes

(10) $$ (c,w) \mapsto c^{\mathrm{T}} C_{Y} D W_{X}^{\mathrm{T}} w.  $$

Since *C*_*Y*_ has orthonormal columns, it is a basis for $\mathbb {R}^{q}$. This implies that *c* is a linear combination of the columns of *C*_*Y*_. We can thus write for *α*=(*α*_1_,…,*α*_*q*_)^T^

(11) $$ c = C_{Y} \alpha, \qquad \alpha^{\mathrm{T}} \alpha = 1,  $$

where the latter identity holds since we require *c*^T^*c*=1. The same holds for *w*=*W*_*X*_*β*, with *β*=(*β*_1_,…,*β*_*p*_) and *β*^T^*β*=1. Now, using the orthogonality of *C*_*Y*_ and *W*_*X*_, we can see that

(12) $$ c^{\mathrm{T}} C_{Y} D W_{X}^{\mathrm{T}} w = \alpha^{\mathrm{T}} D \beta = \sum_{j=1}^{p} \alpha_{j} \beta_{j} d_{j,j},  $$

since *d*_*i*,*j*_=0 for all *i*≠*j*, where *i*=1,…,*q* and *j*=1,…,*p*. Suppose without loss of generality that *p*≤*q*. We can increase the dimensionality of *β* from *p* to *q*, by adding *q*−*p* zeros without changing the unit norm property:

(13) $$ \tilde{\beta} = \left[\beta^{\mathrm{T}},0,\ldots,0\right]^{\mathrm{T}}.  $$

Note that if *q* were to be smaller than *p* then we can use the same argument for *α*. Cauchy-Schwartz tells us that

(14) $$ \begin{aligned} \sum_{j=1}^{p} \alpha_{j} \beta_{j} & = \sum_{i=1}^{q} \alpha_{i} \tilde{\beta}_{i} \\ & = \alpha^{\mathrm{T}} \tilde{\beta} \\ & \leq ||\alpha||\,||\tilde{\beta}|| \\ & = 1 \end{aligned}  $$

The maximum of the covariance (9) is attained only if *α*_1_=*β*_1_=±1. In that case all summands in (12) are zero except when *i*=1, yielding the maximum to be the first (and largest) singular value. The first singular vectors *c*=*C*_*Y*;1_ and *w*=*W*_*X*;1_ are the maximizers. Note that *c*=−*C*_*Y*;1_ and *w*=−*W*_*X*;1_ would also yield equivalently the maximum, this is a minor identifiability problem which does not alter the O2PLS model fit. To get the second direction vectors, we optimize the objective function (9) over the unit norm vectors *c* and *w*; we require also that *c*^T^*C*_*Y*;1_=*w*^T^*W*_*X*;1_=0. This last restriction, the orthogonality constraint, on *c* and *w* imply that *α*_1_=*β*_1_=0 in (12). The maximal covariance is then attained only if |*α*_2_|=|*β*_2_|=1, yielding *c* and *w* to be the second singular vectors *C*_*Y*;2_ and *W*_*X*;2_. Continuing this argument we find the singular vectors in *C*_*Y*_ and *W*_*X*_ to be the maximizers of (9) satisfying the unit norm and orthogonality constraint. If we have a set of indices *I* for which *d*_*i*,*i*_=*d*_*j*,*j*_ for all *i*,*j*∈*I*, we choose $c=C_{Y;\min (I)}$ and $w=W_{X;\min (I)}$ as maximizer. If we have more of those sets, we choose the maximizer in each set in the same fashion.

The orthogonal components are obtained by finding maximal ‘overlap’ between the uncorrected scores *T* and the residuals *E*=*X*−*TW*^T^. An orthogonal score vector is defined as *t*_*Y*⊥_:=*Ew*_*Y*⊥_ where $w_{\textit {Y}\bot }^{\mathrm {T}} w_{\textit {Y}\bot } =1$. We want to maximize the norm of the covariance between *T* and *t*_*Y*⊥_:

(15) $$ \max_{t_{\textit{Y}\bot}} ||T^{\mathrm{T}} t_{\textit{Y}\bot}||^{2}.  $$

This can be rewritten as

(16) $$ \max_{w_{\textit{Y}\bot}} w_{\textit{Y}\bot}^{\mathrm{T}} E^{\mathrm{T}} T T^{\mathrm{T}} E w_{\textit{Y}\bot}.  $$

To incorporate the constraints $w_{\textit {Y}\bot }^{\mathrm {T}} w_{\textit {Y}\bot } = 1$, we introduce a Lagrange multiplier *λ* we and take the derivative with respect to *w*_*Y*⊥_. We get

(17) $$ E^{\mathrm{T}} T T^{\mathrm{T}} E w_{\textit{Y}\bot} = \lambda w_{\textit{Y}\bot}.  $$

The maximum is obtained if *w*_*Y*⊥_ is the eigenvector of *E*^T^*TT*^T^*E* corresponding to the largest eigenvalue. This is the first left-singular vector of *E*^T^*T*. Together with the constraint that *W*_*Y*⊥_ should have orthonormal columns, we find *W*_*Y*⊥_ to be the matrix with left-singular vectors of *E*^T^*T*. The orthogonal scores can be constructed via *T*_*Y*⊥_=*EW*_*Y*⊥_. The same derivation can be used to find that the maximal covariance between *U*_*X*⊥_:=*FP*_*X*⊥_ and *U*, where *F*=*Y*−*UC*^T^, is obtained if *C*_*X*⊥_ is the collection of left-singular vectors of *F*^T^*U*.

### Simulation study

A simulation study was performed to investigate the performance of the O2PLS loading estimates, *W*, *C*, *P*_*Y*⊥_ and *P*_*X*⊥_. Although Trygg et al. included a simulation study in their paper [6], the exact simulation study design was not clearly presented. Therefore we could not reproduce their simulation results, and the parameters for our simulation study were arbitrarily chosen.

The loading values were chosen from a normal probability density function, this reflected the desired property that some variables are important and some not. We designed two dimensionality conditions for the data: the “low” dimensional design stands for *p*=100 variables in *X* and *q*=50 variables in *Y*. In the “high” dimensional setting *X* contains *p*=500 variables and *Y* contains *q*=250 variables. The scores and noise components were randomly drawn from a normal distribution with zero mean. The variances of the scores and noise were chosen so that they would satisfy a noise level condition: the noise level *α*, the relative amount of noisy variation in the data, could take two values; the value *α*=0.05 corresponds to “little” noise setting, noisy variation accounted for 5 % of the total variation. The value *α*=0.5 mimics “much” noise setting, in this case noise accounted for 50 % of the total variation. More precise, the variances ${\sigma _{E}^{2}}$, ${\sigma _{F}^{2}}$ and ${\sigma _{H}^{2}}$ are defined as follows:

(18) $$\begin{array}{*{20}l} {\sigma_{E}^{2}} &=\frac{\alpha}{1-\alpha}\frac{a {\sigma_{T}^{2}} + n_{X} \sigma_{T_{\textit{Y}\bot}}^{2}}{p}, \end{array} $$

(19) $$\begin{array}{*{20}l} {\sigma_{H}^{2}} &=\frac{\alpha}{1-\alpha} {B_{T}^{2}} {\sigma_{T}^{2}}, \end{array} $$

(20) $$\begin{array}{*{20}l} {\sigma_{F}^{2}} &=\frac{\alpha}{1-\alpha}\frac{a \left({B_{T}^{2}} {\sigma_{T}^{2}}+{\sigma_{H}^{2}}\right) + n_{Y} \sigma_{U_{\textit{X}\bot}}^{2}}{q}. \end{array} $$

The number of samples were *N*=500. As a large number of components is not often seen in practice, we chose the number of joint components to be *a*=1. The same holds for the number of orthogonal components: *n*_*X*_=1, *n*_*Y*_=1. Table 1 shows the chosen parameter values in each case. The number of simulation replicates was 1000. We corrected the ‘sign’ of all estimated loading vectors by multiplying the estimated loading vectors with the sign of the crossproduct with the corresponding true loading vectors, for example: $W^{simul}_{\cdot,j} = \text {sign}\left (\!W^{\mathrm {T}}_{\cdot,j} \hat {W}_{\cdot,j}\!\right)\, \hat {W}_{\cdot,j}$ for all joint components *j*=1,…,*a*.

**Table 1:** Simulation parameter choices. The loading value for variable *i* is the density value of a normal distribution with mean *μ* and standard deviation *σ*, denoted as *N*(*i*;*μ*,*σ*). The noise terms were drawn from a normal distribution with zero mean. The scores were drawn from a standard normal distribution. The variances of the noise terms are such that the expected sum of squares of the noise account for 100*α* % (equal to 5 or 50 %) of the total sum of squares

Parameter	‘Low’-dimensional case	‘higher’-dimensional case
*N*	500	500
*p*,*q*	[100,50]	[500,250]
*W*	[*N*(*i*;60,10)]_*i*=1,…,100_	[*N*(*i*;300,50)]_*i*=1,…,500_
*C*	[*N*(*i*;70,5)]_*i*=1,…,50_	[*N*(*i*;175,25)]_*i*=1,…,250_
*P*_*Y*⊥_	[*N*(*i*;20,20)]_*i*=1,…,100_	[*N*(*i*;100,100)]_*i*=1,…,500_
*P*_*X*⊥_	[*N*(*i*;15,10)]_*i*=1,…,50_	[*N*(*i*;75,50)]_*i*=1,…,250_
*B*_*T*_	2	2
${\sigma _{T}^{2}},\sigma _{T_{\textit {Y}\bot }}^{2},\sigma _{U_{\textit {X}\bot }}^{2}$	[1,1,1]	[1,1,1]
${\sigma _{E}^{2}},{\sigma _{F}^{2}},{\sigma _{H}^{2}}$	$\frac {\alpha }{(1-\alpha)}[0.02,0.104,4]$	$\frac {\alpha }{(1-\alpha)}[0.004,0.021,4]$

Implementation of the O2PLS algorithm, calculations and analyses were conducted in R [9].

### Availability of supporting data

The metabonomic measures are available as Supplementary Table 4 in [5]. The raw and normalized gene expression intensities have been deposited in ArrayExpress which can be found at: http://www.ebi.ac.uk/arrayexpress/ under the accession number E-TABM-1036. ArrayExpress is hosted by the European Bioinformatics Institute.

## Results

### Results of simulation study

**Fig. 2:**
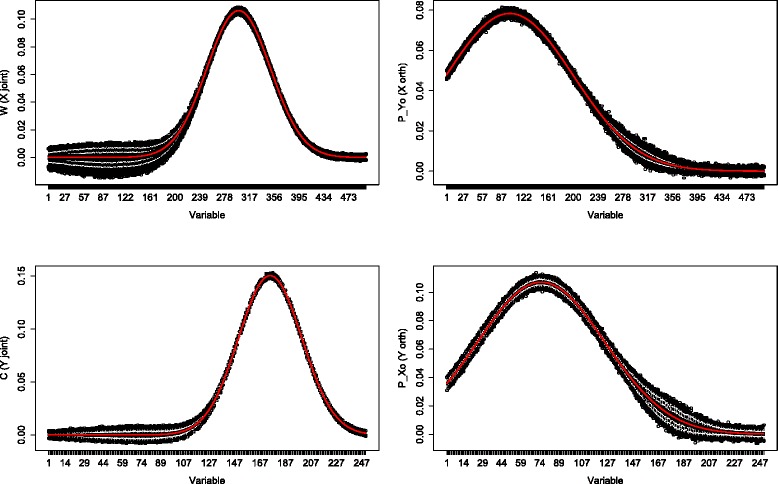
Simulation: high dimensions little noise. Boxplots of 1000 simulations in which *X* (*upper* row) contains 500 samples and 500 variables, *Y* (*lower* row) contains 500 samples and 250 variables. Noise contributed for 5 % of the total variation. The first column corresponds to the joint part, the second column depicts the orthogonal part. The red line denotes the true loading profile

For each loading parameter we obtained 1000 estimates. Boxplots for the joint (left column) and orthogonal (right column) part estimates in *X* (upper row) and *Y* (lower row) in the “little” noise case (*α*=0.05) are shown in Figs. 1 and 2.

Firstly in both “low”(*p*=100, *q*=50) and “higher”(*p*=500, *q*=250) dimensions, the accuracy of the estimates were very similar, as can be seen from the location and range of the boxplots. Secondly at the variables with a high joint loading value but low orthogonal loading value, the orthogonal part estimates followed the true orthogonal loading profiles. The joint part estimates also followed the true joint loading profiles regardless of the value of the orthogonal loadings at those variables. Thirdly, the difference between the estimates for the *X*and *Y* components was minor. There was slightly more variation present in the *X* data at variables with a low loading value.

**Fig. 3:**
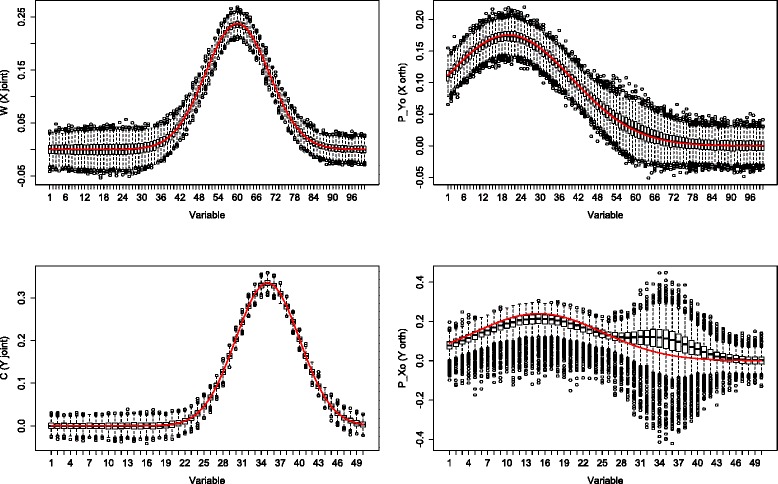
Simulation: low dimensions high noise. Boxplots of 1000 simulations in which *X* contains 500 samples and 100 variables, *Y* contains 500 samples and 50 variables. Noise contributed for 50 % of the total variation. The first column corresponds to the joint part, the second column depicts the orthogonal part. The red line denotes the true loading profile

**Fig. 4:**
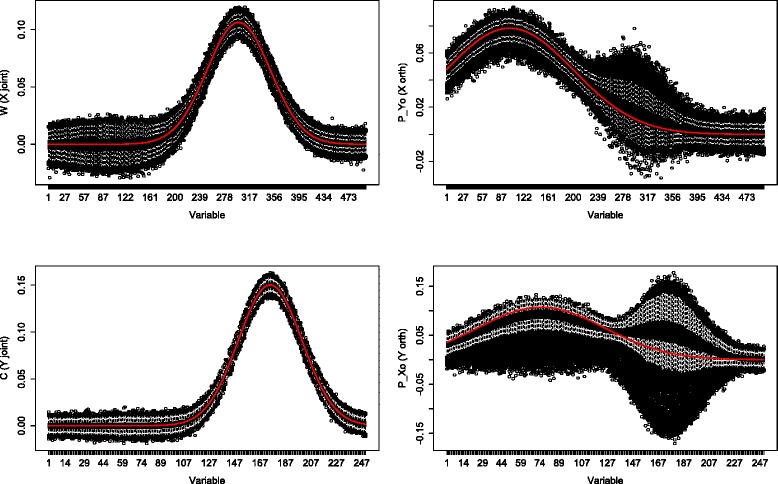
Simulation: high dimensions high noise. Boxplots of 1000 simulations in which *X* contains 500 samples and 500 variables, *Y* contains 500 samples and 250 variables. Noise contributed for 50 % of the total variation. The first column corresponds to the joint part, the second column depicts the orthogonal part. The red line denotes the true loading profile

Boxplots of the 1000 simulations for the “much” noise case (*α*=0.5) are shown in Figs. 3 and 4. In both “low”(*p*=100, *q*=50) and “higher”(*p*=500, *q*=250) dimensions the estimates performed similar. The joint part estimates still followed the true loading profile, although the boxplots showed more variation across the 1000 estimates. The orthogonal part estimates were less accurate than the orthogonal part estimates in the “low” noise case. Especially at the variables with a high joint loading value, the orthogonal part estimates showed a high variation. The orthogonal part estimates in *Y* were visibly higher in at least 75 % of the simulation replicates. When simulating similar sizes as in our data example (we took *p*=6000 and *q*=140 and considered *α*=0.5), the O2PLS method showed the same behavior (not shown).

### Application to DILGOM data

Samples on metabolome (137 variables) and transcriptome (35,419 variables) were collected as part of the ‘Dietary, Lifestyle, and Genetic determinants of Obesity and Metabolic syndrome’ (DILGOM) study [5]. Study participants were aged 25–74 years, median age was 53, and were sampled from the region of Helsinki, Finland. A total of 506 participants were present in both studies, of which 232 male and 274 female. In this analysis, we excluded participants whenever they had a missing value for one or more measurements in either the metabolomics or transcriptomics data. This resulted in 40 omitted participants, the used data thus finally consisted of *N*=466 participants.

The metabolomics data were derived from nuclear magnetic resonance (^1^H NMR), providing absolute quantitative measurements on the serum metabolome. The transcriptomics data were derived from averaged gene expression counts on technical replicates. The raw counts were quantile normalized at strip level. For more detailed info, see [5, 10]. In transcriptomics filters are proposed to reduce the amount of uninformative (low variance and expression level) variables, which are often interpreted as containing noise. The original study [5] used a filter retaining only the 10 % highest expression levels, and considered 3520 gene expression variables for analysis. To model the orthogonal noise components we were less stringent and extracted the top 25 % of the absolute values of the gene expressions, and we intersected this set of expressions with the set containing the 25 % expressions with the largest inter-quantile range conform [11]. The reduced transcriptomics data contained 6272 variables. Results of the analysis with all 35,419 variables were very similar (not shown).

A Box-Cox transformation [12] with parameter $\frac {1}{4}$ was performed for the metabolomics data, to reduce skewness. The ‘best’ choice for the Box-Cox parameter has been investigated by many, we observed from the first four central moments that $\frac {1}{4}$ was sufficient to continue the data analysis. Inouye et al. [5] also applied a Box-Cox transformation per variable, but the powers of the transformations were not stated. A scaling here would amplify the effect of noise on the estimates, so the data were only mean centered.

**Fig. 5:**
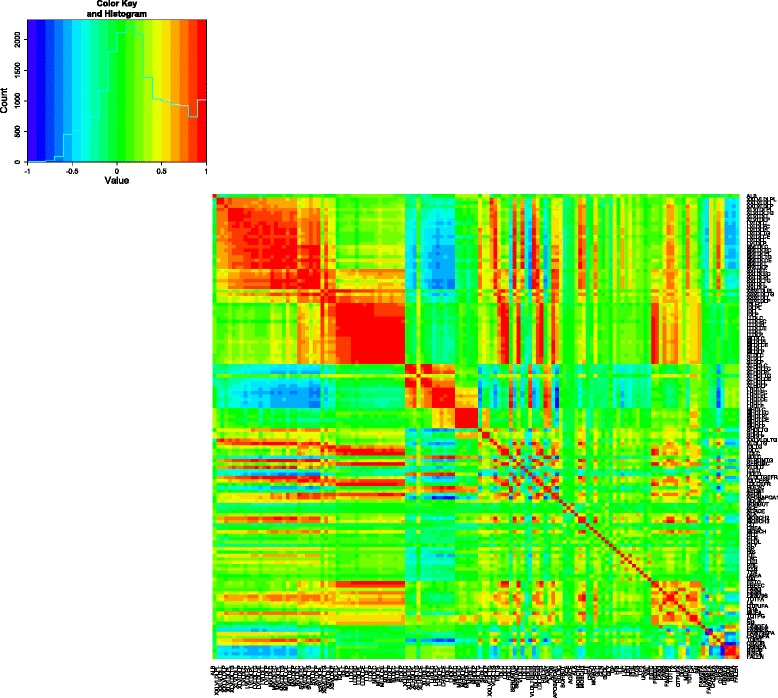
Pearson correlation heatmap of metabolites. Red indicates high positive correlation, green is little correlation and blue is high negative correlation. The variables are in the original order. A histogram of correlations is added in the top left corner

To give an overall impression, the pairwise Pearson correlation coefficients between the metabolite variables are depicted in a heatmap in Fig. 5. There was a cluster of positively correlated variables present within the various lipoproteins (VLDL, LDL, IDL, HDL) subgroups. The VLDL subgroup and the HDL subgroup had negative correlation. Due to the large number of variables in the transcriptome data, a heatmap of the correlations the variables is omitted.

We continued our data analysis with the integration of metabolomics (*X*) and transcriptomics (*Y*), using O2PLS. To determine the optimal number of components, we utilized the proposed alternative cross-validation procedure as discussed in Section “Methods”, initializing with *a*=1,2,…,10. The optimal number of model components were found *a*=1, *n*_*X*_=1, *n*_*Y*_=8. The modeled variations per component is shown in Table 2. In terms of explained variances (*R*^2^) we observed the following:

- The variation in *X* and *Y* explained by the model was 58 and 51 % respectively. The rest of the variation was estimated as noise.

**Table 2:** Absolute and relative variations in O2PLS

*a*	${R^{2}_{X}}$	${R^{2}_{Y}}$	$R^{2}_{\text {Xcorr}}$	$R^{2}_{\text {Xcorr}}$	$R^{2}_{\text {Xhat}}$	$R^{2}_{\text {Yhat}}$	$R^{2}_{\text {Xhat}}$/$R^{2}_{\text {Xcorr}}$	$R^{2}_{\text {Yhat}}$/$R^{2}_{\text {Ycorr}}$
1	57.97	50.81	46.31	1.37	26.74	0.80	57.74	58.55
2	67.94	53.40	60.80	4.24	29.52	1.45	48.55	34.25
3	74.08	54.79	68.99	7.35	26.70	2.00	38.69	27.23
4	78.06	55.62	72.94	9.63	29.23	2.40	40.07	24.87
5	80.93	56.69	76.51	11.30	29.81	3.32	38.97	29.43
	$1-\frac {\sum {E_{i,j}^{2}}}{\sum X_{i,j}^{2}}$	$1-\frac {\sum {F_{i,j}^{2}}}{\sum Y_{i,j}^{2}}$	$\frac {\sum \left (TW^{\mathrm {T}}\right)_{i,j}^{2}}{\sum X_{i,j}^{2}}$	$\frac {\sum \left (UC^{\mathrm {T}}\right)_{i,j}^{2}}{\sum Y_{i,j}^{2}}$	$\frac {\sum \left (UB_{U}W^{\mathrm {T}}\right)_{i,j}^{2}}{\sum X_{i,j}^{2}}$	$\frac {\sum \left (TB_{T}C^{\mathrm {T}}\right)_{i,j}^{2}}{\sum Y_{i,j}^{2}}$		

The amount of variation per model statistic with respect to the total amount of variation, from an O2PLS fit using Metabolomics (*X*) and Transcriptomics (*Y*). The *R* ^2^ (definition given in last row) in *percentages* (with respect to the total variation in *X* and *Y* respectively) for each model statistic. The numbers of orthogonal components are *n*_*X*_=1,*n*_*Y*_=8. The number of joint components varies from 1–5. The first row was found best according to the proposed alternative cross-validation (as in Section “Methods”)
- The joint correlated part in *X* explained 46 % of the variation in *X*. Further 1 % of the total variation in *Y* was explained by the joint correlated part in *Y*. This means that 46 % of *X* and 1 % of *Y* could be explained with one another.
- Of the 46 %, *Y* explained 27 % of *X*. This could be seen relatively as 57 % of the joint variation in *X*. Furthermore 0.8 % of *Y* was explained by *X*, which was 58 % of the explainable variation in *Y*.

The sum of squares of all scores in the fitted model are given in Table 3. The orthogonal part in *Y* explains about half of the variation in *Y*, while half of the variation in *X* is explained by the joint part. This is due to the larger number of components in the orthogonal part in *Y*. About 50 % of the total variation is due to noise.

**Table 3:** Absolute and relative variations of the scores and noise in O2PLS

	*T*	*T*_*Y*⊥_	*E*	*U*	*U*_*X*⊥_	*F*	*H*
Absolute	2551	642	2316	3852	138502	137837	2061
Relative	46.3 %	11.7 %	42.0 %	1.4 %	49.4 %	49.2 %	53.5 %

The sum of squares per model part in an O2PLS fit using Metabolomics (*X*) and Transcriptomics (*Y*). Absolute quantities as well as percentages with respect to the total variation in *X* (first three), *Y* (second three) and *U* (last one) are shown

**Fig. 6:**
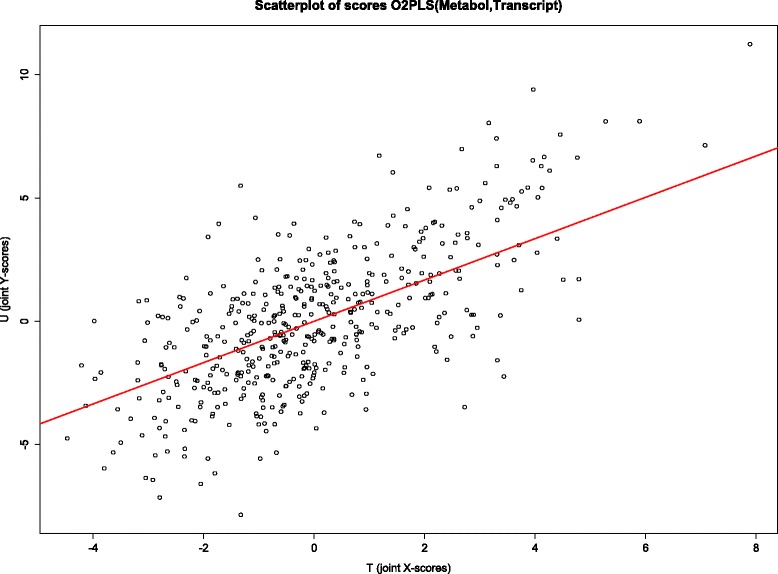
Scatterplot joint score vectors. The first joint score vectors (*T*, *U*) obtained from an O2PLS fit using Metabolomics (represented by *T*) and Transcriptomics (represented by *U*) are plotted against each other. The slope of the fitted line is 0.84, the intercept is zero due to the mean centering of the data. The coefficient of determination *R* ^2^ was 0.47

Next in order to evaluate the quality of predictions of *Y* with *X*, a scatter plot of *U* versus *T* is given in Fig. 6. The slope of the regression line equaled *B*_*T*_=0.84. The *R*^2^ of the regression of *U* on *T* was 0.47.

**Fig. 7:**
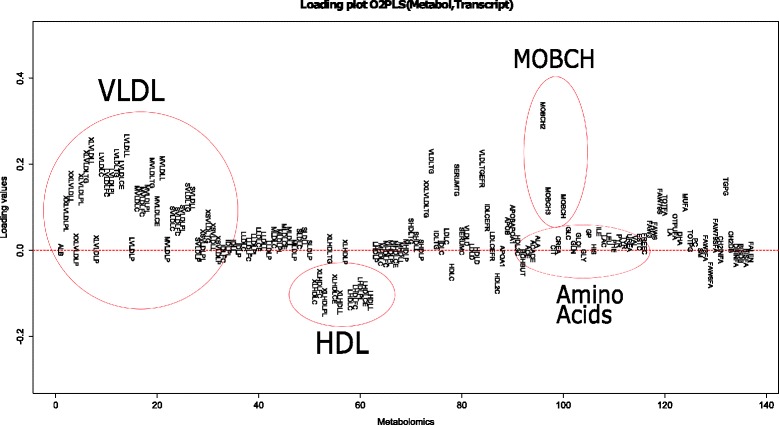
Labeled joint metabolomic loading plot. Four groups of interest are grouped: very-low-density-lipoproteins, high-density-lipoproteins, mobile lipids and amino acids

**Fig. 8:**
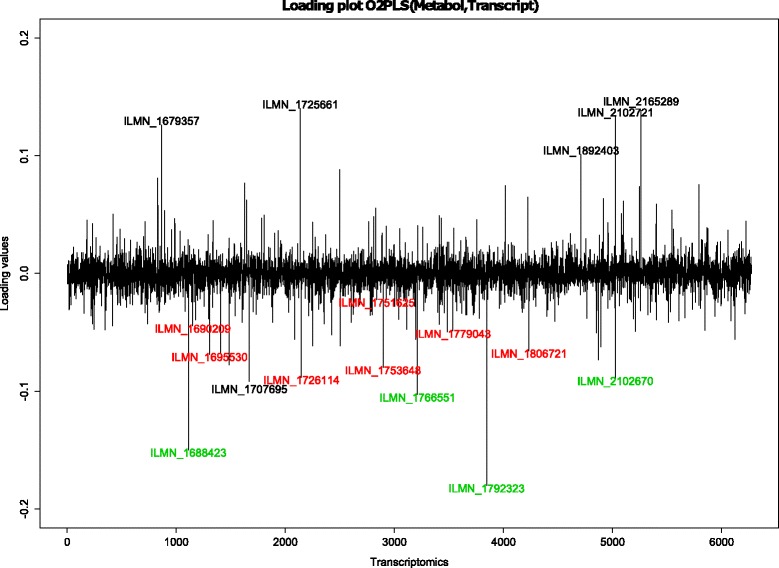
O2PLS transcriptomic joint loadings. Joint part O2PLS loadings per gene expression. The top ten gene expressions are in black and green. The LL module gene expressions are in red and green. Four of the eleven gene expressions in the LL module are in the top ten, indicated in green. The loadings for five other gene expressions in the top ten and the loadings for the LL module gene expressions have opposite sign

In the light of Inouye’s results [5], the role of the LL module (a cluster of tightly correlated co-expressed genes) in metabolic variation was analyzed with O2PLS. The gene expression labels and corresponding genes are shown in Table 4. Figure 7 shows the estimated joint loading values for each metabolite (overall mean 0.0363). The VLDL subgroup together with MOBCH2-MOBCH3 had large estimated loadings (mean 0.116, max 0.314). The HDL subgroup was estimated to have moderate loading values (mean –0.0439, min –0.121), note that the loading values were negative. This coincides with the negative correlation between VLDL and HDL. The magnitude of the loading values for the other lipoprotein subgroups were small, and approximately proportional to their size (mean 0.0171, max 0.0763). In Fig. 8 the estimated joint loadings for the gene expression variables are shown (overall mean –0.000350). There are some variables noticeable for their estimated loading size: For the top 10 gene expressions the ID label was shown next to their estimates in black. The LL module gene expressions were labeled in the plot using a red color. For LL module gene expressions in the top 10, the color green was used. The labels and corresponding genes are shown in Table 5. The two gene expressions with the highest absolute loading values were also in the LL module (loading values −0.180 and −0.150 respectively).

**Table 4:** Gene composition of the LL module identified by Inouye et al.

Gene annotation	Ilumina ID
C1ORF186	ILMN_1690209
CPA3	ILMN_1766551
ENPP3	ILMN_1749131
FCER1A	ILMN_1688423
GATA2	ILMN_2102670
HDC	ILMN_1792323
HS.132563	ILMN_1899034
MS4A2	ILMN_1806721
SLC45A3	ILMN_1726114
SPRYD5	ILMN_1753648
CACNG6	ILMN_1779043

**Table 5:** LL module and top 10 gene expressions. Identified gene expressions in the top 10 most important variables for the joint variation in the transcriptome. The corresponding genes are shown. Four gene expressions fall into the earlier identifies Lipid-Leukocyte module

Gene annotation	Ilumina ID	Module
CPA3	ILMN_1766551	LL and top 10
FCER1A	ILMN_1688423	LL and top 10
GATA2	ILMN_2102670	LL and top 10
HDC	ILMN_1792323	LL and top 10
DEFA1B	ILMN_1725661	top 10
DEFA1B	ILMN_1679357	top 10
DEFA1B	ILMN_2102721	top 10
SNORD13	ILMN_1892403	top 10
DEFA3	ILMN_2165289	top 10
IFIT1	ILMN_1707695	top 10

**Fig. 9:**
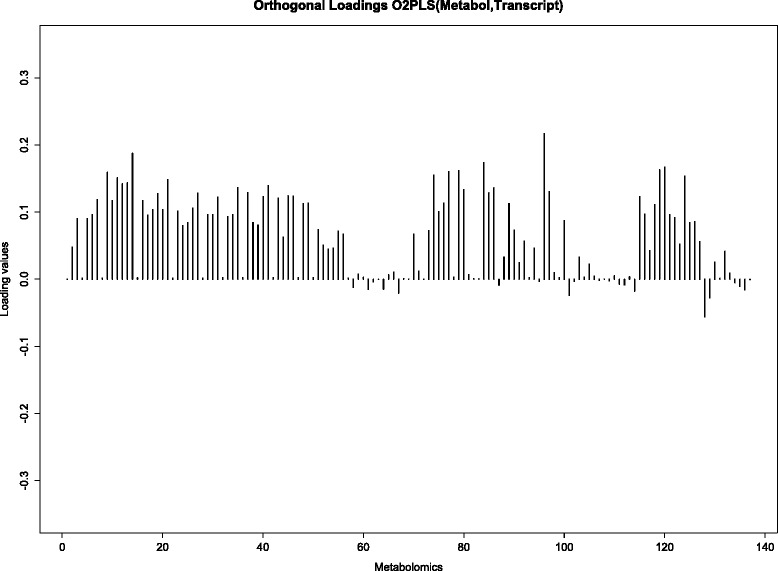
O2PLS metabolomic orthogonal loadings. Orthogonal part loadings obtained from an O2PLS fit with Metabolomics and Transcriptomics. One orthogonal component in metabolomics was identified

**Fig. 10:**
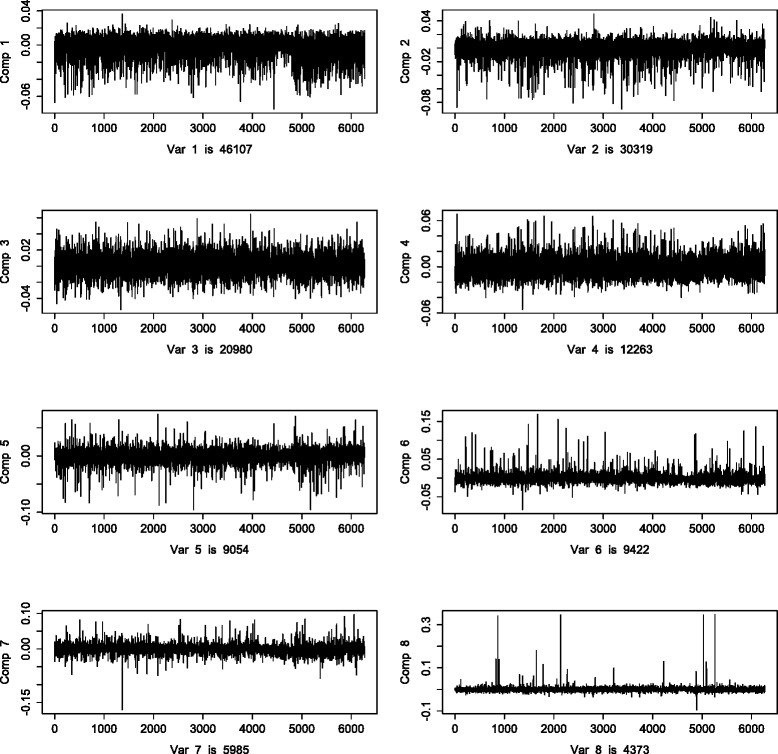
O2PLS transcriptomic orthogonal loadings. Orthogonal part O2PLS loadings per gene expression. There were eight orthogonal components identified. The ratio of the first part sum of squares and last part sum of squares is approximately eleven

One orthogonal component was identified in the metabolomic data. The loading vector, which is normed to one, is shown in Fig. 9. The metabolomic orthogonal loading values are less diverse than the joint loading values. The HDL subgroup and amino acids got small absolute loading values, the other metabolites had an equal share in the orthogonal variation. There were eight orthogonal components identified in the transcriptomics data. For comparison purposes, the loading vectors were orthonormalized. The eight loading vectors, together with the variation per component, are plotted in Fig. 10. Note that different loading values across components cannot directly be compared, since the variations are not equal. The first loading vectors show little structure. In the last plot we can see few large peaks, indicating that only some variables are of importance in that component. The variation in the first component is approximately eleven times larger than the variation in the last component.

## Discussion

The integrative systems biology approach is becoming increasingly popular and integration of omics data will provide more insight into the biological systems. The PLS method is widely known in chemometrics and provides data integration and simultaneous modeling, but as shown in [6] the estimates are sensitive to structural noise. While OPLS [7] provides correction for such orthogonal variation, it is oriented towards predicting an outcome and thus lacks symmetry. We considered here the O2PLS method [6]; it is a symmetric data integration method, accounting for structural noise in both matrices. We particularly aimed to integrate two omics data sets for embedding a high dimensional data set in terms low dimensional ‘latent’ variables. To extract relevant information in the data sets, we decompose the two data sets into three parts: joint part in which variables in one data set are related to those in another data set; orthogonal part in which variables are not related, but still important, in each of the data sets; and noise. Simultaneously we searched for the relevant variables in each part.

Several approaches similar to O2PLS are available. To handle more than two data sets, a generalization of O2PLS has been proposed in [13], called OnPLS. Methods to deal with the general idea of decomposing data sets in a joint and systematic part have been proposed. They differ in methodology and estimation. For example, DISCO-SCA [14] can handle multiple data sets and may perform better when prior information about the configuration of the joint and orthogonal components is available. An essential assumption in this model is that the components scores or loadings in each data set are exactly the same. Another method providing data decomposition in a joint and orthogonal part is JIVE [15], which can also handle more than two data sets. JIVE may be used if the common source underlying all data sets are similar/homogeneous. One should note that that JIVE restricts the joint part to be orthogonal to the systematic parts. Though it may be argued that the joint and systematic loadings in the population are orthogonal, when obtaining a sample from this population the joint and systematic loadings will typically not be orthogonal. This orthogonality of the joint and systematic loadings is not essential in O2PLS. More research is needed to assess the impact of these methods.

A simulation study is conducted to assess the accuracy of the O2PLS estimates, see Figs. 1, 2, 3 and 4. The estimates were accurate if “little” noise was present (proportion of noise in the data is *α*=0.05). The model can distinguish well between joint and orthogonal variation. This is the case in both “low”(*p*=100, *q*=50) and “higher”(*p*=500, *q*=250) dimensional simulated data. The presence of “much” noise (*α*=0.5) did not cause a substantial decrease in accuracy of the joint part estimates. They followed the true underlying loading profile well. The orthogonal part estimates were affected by more noise in a negative way. Especially in the “higher” dimensional case, the orthogonal part estimates concerning *Y* (*q*=250) are biased upwards. The model cannot distinguish well joint and orthogonal variation, it mixes up both loading profiles. It may be argued that the estimation method of the joint loadings is borrowing accuracy from both two data sets, while the orthogonal loadings estimation method is less precise since it uses noisy remaining (total minus joint) variation. Similar to any method, under noisy circumstances it will be difficult to estimate the true orthogonal loadings. This effect was less in the orthogonal part in *X* (*p*=500), which has higher dimensions. It is not clear why the orthogonal part estimates with less parameters (the orthogonal part in *Y*) degrade more than those with more parameters (the orthogonal part in *X*) in the presence of noise.

We integrate data on the metabolome and transcriptome, extracting both the joint and the orthogonal part, provided in the O2PLS fit, in both data sets. Finding the optimal number of components is a computationally expensive task. A balance between computation time and accuracy is sought by maximizing the explained variance in the inner relation to determine the number of orthogonal parts, and then minimizing the prediction error for determining the number of joint parts. Investing more time in this particular subject will aid in choosing a more accurate method, without compromising computational efficiency. We find four of the eleven LL module gene expressions among the top ten, in terms of importance for the joint variation (Fig. 8). Moreover, the two gene expressions with the highest absolute loading values are in the LL module. Furthermore in the metabolomics data we find the VLDL subgroup together with the HDL subgroup to be important for the joint variation in the metabolomics data (Fig. 7). This shows a contribution of the LL module to the joint variation, partially induced by the VLDL and HDL subgroups. This result can be found back in [5]. The simultaneous data analysis approach identifies more expressed genes important for the joint variation, the ID’s are in Table 5. All genes except SNORD13 are involved in immune/defence system pathways, but information for SNORD13 is at the time of writing unavailable. Also there is large contribution from the mobile lipids MOBCH2 and MOBCH3 to the joint metabolite variation. The orthogonal variation in this data is difficult to interpret, no noticeable trends or clusters were found in the loading values (Figs. 9 and 10). Including orthogonal components in the model does improve the cross-validated prediction error (which depends on the joint components), which makes it still useful to include in the model. As we saw from the simulation results in the“higher” noise (50 %) case (the estimated amount of noise in the metabolomics and transcriptomics data is also around 50 %), the joint loading estimates still follow the profile of the true loadings. The orthogonal loading estimates are performing worse, indicating a loss of accuracy and thus interpretation in the orthogonal components.

To meet the challenge of interpretation of the results and to infer the relative importance of the variables a structured and tractable probabilistic framework is required. It is beyond the scope of this paper to propose a new method; nevertheless, we argue for the necessity and the feasibility of such a framework. Due to a lack of an explicit probabilistic model in O2PLS, it is not straightforward how to perform statistical tests on the loadings. For PLS, a bootstrap approach is proposed in [16]. In the O2PLS model we must take into account the orthogonal loadings, which are correlated with the joint loadings due to the nature of the estimation algorithm. This may invalidate the bootstrap results. Furthermore a potential problem of multiple testing may exist, which needs to be correctly addressed. The assumptions made in the model imply that the orthogonal scores *T*_*Y*⊥_ and *U*_*X*⊥_ cannot be seen as realisations of random variables, which is a fundamental property in statistical inference. Furthermore without additional assumptions on the orthogonal part loadings *P*_*Y*⊥_ and *P*_*X*⊥_ the model is unidentifiable. Also, the probabilistic approach gives insight in hidden flaws of the estimators, which are very difficult to discover with the current O2PLS algorithm. These potential problems may invalidate statistical inference on the whole population.

Providing a probabilistic framework to non-probabilistic methods was done earlier. Probabilistic PCA has been developed in [17], and for the factor analysis model there is a well written probabilistic approach in [18]. A novel probabilistic approach for the O2PLS method, which puts the O2PLS method in a statistical framework, is currently being developed. The optimization criterion will be maximum likelihood. The use of a parametric model and a likelihood are indeed restricting the researcher, as one needs to assume a distribution on the data. However we expect that the probabilistic O2PLS model, just as the ordinary linear model, will be robust against small violations of the assumptions. The resulting likelihood can be easily optimized, using a factorization of the probability density which allows for seperately optimizing the likelihood.

A new derivation in multiplatform data analysis we intend to do is the use of a likelihood information score, which will rely on PO2PLS, indicating how much or little two data sets are related. Combining the data integration approach with a probabilistic framework will aid interpretability and inference in more general epidemiological studies.
